# P-513. Recruitment Challenges for Youth HIV Pre-exposure Prophylaxis (PrEP) Research

**DOI:** 10.1093/ofid/ofae631.712

**Published:** 2025-01-29

**Authors:** Lisa Abuogi, Alexander Limas, Jennifer Moor, A J De La Garza, Katherine Leonard, Kimberly Pierce, Sam Gallegos, Priya H Vyas, Elizabeth McFarland, Diane M Straub

**Affiliations:** University of Colorado, Denver, Colorado; University of Colorado, Denver, Aurora, Colorado; University of Colorado Denver, Aurora, Colorado; University of Colorado Denver, Aurora, Colorado; University of Colorado Denver, Aurora, Colorado; University of Colorado Denver, Aurora, Colorado; University of Colorado Denver, Aurora, Colorado; University of Colorado Denver, Aurora, Colorado; University of Colorado Denver, Aurora, Colorado; University of Colorado Denver, Aurora, Colorado

## Abstract

**Background:**

Recruitment for youth-focused PrEP studies among sexual and gender minority (SGM) youth is a challenge. We report on recruitment strategies and lessons learned from a youth TelePrEP study in Colorado.

**Table 1:**
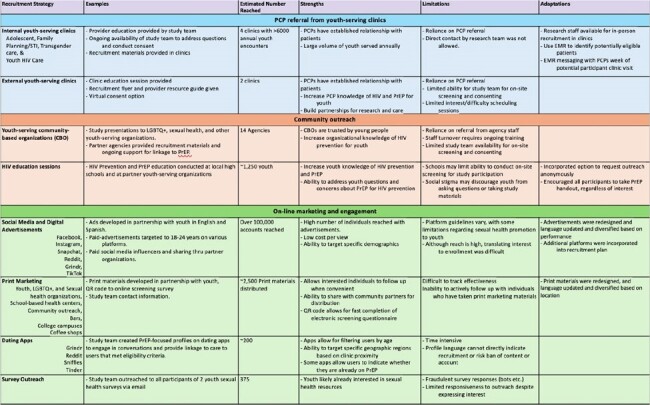
Recruitment strategies used for youth PrEP study

**Methods:**

Youth assigned male at birth, ages 18-24 years, eligible for PrEP using FTC/TAF, and at risk for HIV in Colorado were recruited to participate in a pilot feasibility trial of PrEP services provided via telehealth. Participants agreed to be seen via telehealth and to obtain initial lab testing at one of seven sites located throughout the state. PrEP medications were offered free for up to 12 months via in-person pick up or mail delivery. Youth were able to enroll and receive clinical care regardless of insurance type. HIV self-test kits were available via mail for follow up testing. Participants received $25 at time of enrollment. Recruitment methods were informed by two electronic youth surveys (N= 375 total), student young adult advisors, youth PrEP providers, and research team experience in youth HIV treatment and prevention studies.

**Figure 1:**
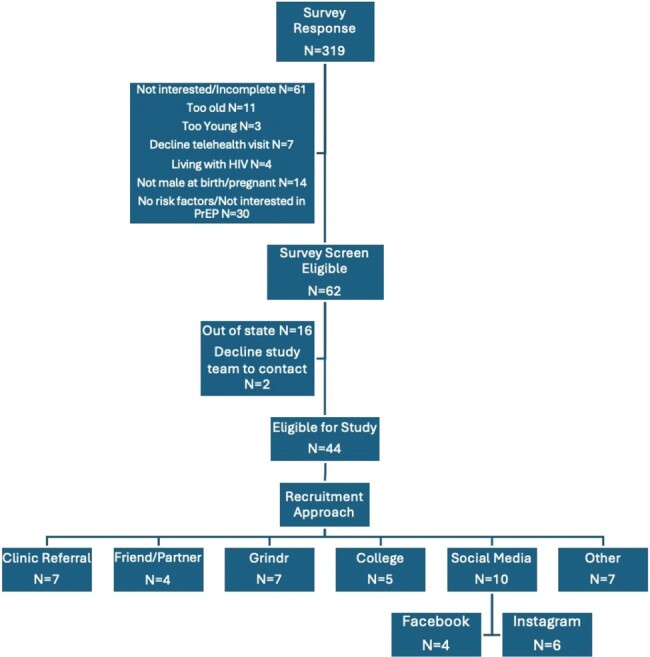
Recruitment Flow Chart

**Results:**

A total of 319 individuals completed an online recruitment survey of which 44 (14%) were eligible and 21 (48%) of those enrolled between January 2023 and March 2024 out of a target of 100 study participants. (Figure 1) An estimated 107,500 individuals received at least one form of study recruitment. (Table 1) Direct recruitment occurred through adolescent clinics (including family planning/STI, transgender care, adolescent primary care, and youth HIV clinics) and pediatric primary care offices via primary care provider referral (N=4 enrolled). Community recruitment was done through SGM youth-serving organizations, community events, during community HIV education sessions (N=4 enrolled). On-line recruitment utilized social media (including targeted ads and sponsored influencers), dating apps, and survey outreach (N=6 enrolled). (Figure 2) Dating apps and social media were the most common recruitment strategies among eligible participants.

**Figure 2:**
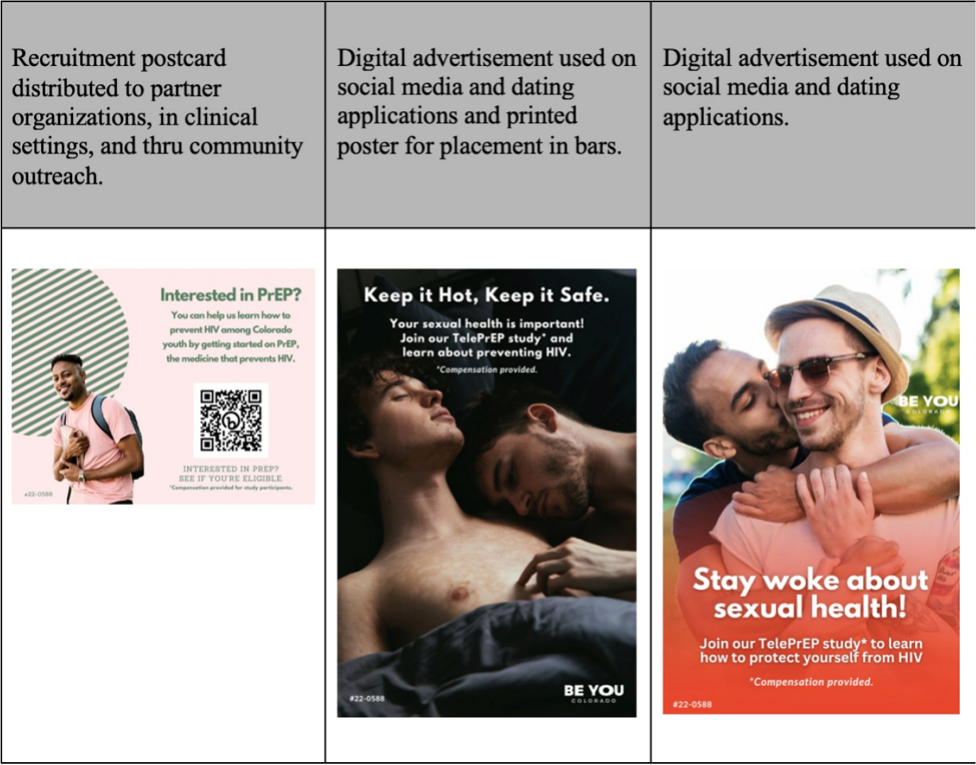
Examples of Youth TelePrEP Recruitment Materials

**Conclusion:**

Despite a multipronged approach to recruitment of SGM youth for a study on youth TelePrEP, recruitment was below targets. Additional innovation and evaluation of strategies are needed to not only increase recruitment to youth PrEP studies but also youth PrEP services.

**Disclosures:**

**Lisa Abuogi, MD, MS**, Gilead Sciences: Grant/Research Support **Alexander Limas, MSW**, Gilead Sciences: Grant/Research Support **AJ De La Garza, BSW**, Gilead Sciences: Grant/Research Support **Katherine Leonard, MSN, FNP-BC**, Gilead Sciences: Grant/Research Support **Sam Gallegos, BS**, Gilead Sciences: Grant/Research Support

